# Space-related pharma-motifs for fast search of protein binding motifs and polypharmacological targets

**DOI:** 10.1186/1471-2164-13-S7-S21

**Published:** 2012-12-07

**Authors:** Yi-Yuan Chiu, Chun-Yu Lin, Chih-Ta Lin, Kai-Cheng Hsu, Li-Zen Chang, Jinn-Moon Yang

**Affiliations:** 1 Institute of Bioinformatics and Systems Biology, National Chiao Tung University, Hsinchu, 30050, Taiwan; 2 Department of Biological Science and Technology, National Chiao Tung University, 75 Po-Ai Street, Hsinchu, 30050, Taiwan

## Abstract

**Background:**

To discover a compound inhibiting multiple proteins (i.e. polypharmacological targets) is a new paradigm for the complex diseases (e.g. cancers and diabetes). In general, the polypharmacological proteins often share similar local binding environments and motifs. As the exponential growth of the number of protein structures, to find the similar structural binding motifs (pharma-motifs) is an emergency task for drug discovery (e.g. side effects and new uses for old drugs) and protein functions.

**Results:**

We have developed a Space-Related Pharmamotifs (called SRPmotif) method to recognize the binding motifs by searching against protein structure database. SRPmotif is able to recognize conserved binding environments containing spatially discontinuous pharma-motifs which are often short conserved peptides with specific physico-chemical properties for protein functions. Among 356 pharma-motifs, 56.5% interacting residues are highly conserved. Experimental results indicate that 81.1% and 92.7% polypharmacological targets of each protein-ligand complex are annotated with same biological process (BP) and molecular function (MF) terms, respectively, based on Gene Ontology (GO). Our experimental results show that the identified pharma-motifs often consist of key residues in functional (active) sites and play the key roles for protein functions. The SRPmotif is available at http://gemdock.life.nctu.edu.tw/SRP/.

**Conclusions:**

SRPmotif is able to identify similar pharma-interfaces and pharma-motifs sharing similar binding environments for polypharmacological targets by rapidly searching against the protein structure database. Pharma-motifs describe the conservations of binding environments for drug discovery and protein functions. Additionally, these pharma-motifs provide the clues for discovering new sequence-based motifs to predict protein functions from protein sequence databases. We believe that SRPmotif is useful for elucidating protein functions and drug discovery.

## Background

During the early drug discovery stage, discovering potential target proteins for a compound is important to drug design and to reduce cost and time by detecting the potential harmful side effects. On the other hand, it could provide the new usages for old drugs. Recently, to design a drug inhibiting multiple target proteins (i.e. polypharmacological targets) builds a new paradigm for diseases with complex mechanisms, such as cancers and diabetes. Therefore, discovering polypharmacological targets, share similar interfaces and are often inhibited by the same compound, is a valuable issue in understanding binding mechanisms and drug development.

To identify the proteins with similar binding sites of a given protein sequence or structure by searching the sequence or structure databases has usually used [1-4]. In general, the proteins (i.e. polypharmacological targets) bound the same ligand should share similar binding environments, but they may not have significant evolutionary relationship in both sequences and global structures. As the exponential growth of the number of protein structures, protein structures have been proposed to analyze the structural motifs and to describe the binding environment [5,6]. Most of these studies (e.g. SPASM [7], Superimpose [8], RASMOT-3D PRO [9], MultiBind [10], and Wu *et al. *[11]) search the similar local structures or binding sites (active sites) based on only one structural motif. However, a protein-ligand binding interface usually consists of a set of spatially discontinuous structural motifs. For example, two motifs (i.e. HxGH loop and KMSKS loop) catalyse the amino acid activation with ATP in class-I aminoacyl-transfer RNA synthetases [12].

To address these issues, we proposed Space-Related Pharmamotif (SRPmotif) method to identify the pharma-interfaces (≥ 2 structural motifs) sharing similar binding environments from the polypharmacological targets. The pharma-interface consists of a set of spatially discontinuous peptide segments (i.e. pharma-motifs), which surround the ligand-binding site. By transforming the 3D structure segments into 1D structural alphabet sequences through in-house tool (called 3D-BLAST [13,14]), we can rapidly search the potential target proteins with similar binding environment against Protein Data Bank (PDB) [15]. And then a structural alignment tool, such as DALI [16], was used to precisely locate the possible binding environment in these protein structures. Experimental results showed that around 56% interacting residues were considered as highly conserved in our identified pharma-motifs. These conserved residues involving in pharma-motifs often play the key roles for biological functions and binding mechanisms. Furthermore, these pharma-motifs can be transferred into sequence-based motifs for searching similar proteins with local binding-site similarity from protein sequence databases. We believe that SRPmotif is useful for studying protein functions and discovering the polypharmacological targets for drug design. The SRPmotif is available at http://gemdock.life.nctu.edu.tw/SRP/.

## Materials and methods

### Pharma-motifs and pharma-interfaces

**Figure 1:**
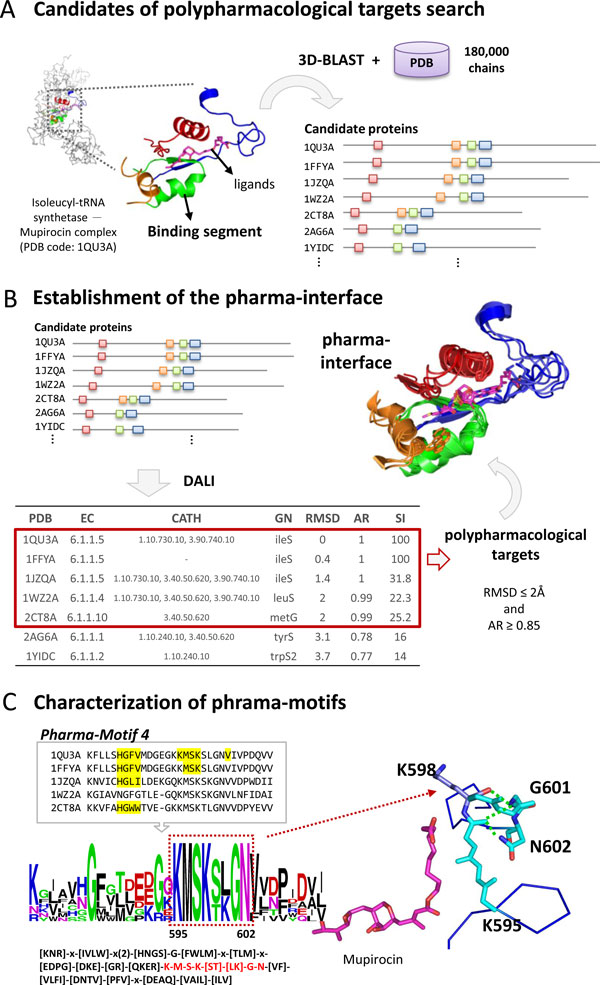
**Overview of SRPmotif using Isoleucyl-tRNA synthetase-Mupirocin complex as example**. (A) Identify polypharmacological candidates with similar discontinuous binding segments by rapidly searching protein structure databases using 3D-BLAST. The proteins with 75% similar binding segments are considered as the candidates of the polypharmacological candidates. (B) These candidates with significantly similar interfaces (RMSD ≤ 2.0Å and average aligned ratio ≥ 0.85) are considered as polypharmacological proteins. (C) The polypharmacological proteins are used to recognize the conserved segments (pharma-motifs) which can be transformed into 1D sequence patterns based on the multiple structural alignments. These spatially discontinuous pharma-motifs are formed a pharma-interface for these polypharmacological proteins.

Here, we proposed a new concept and a fast method (SRPmotif) to identify space-related pharma-motifs and pharma-interfaces for discovering polypharmacological targets. We considered a pharma-interface as a conserved binding environment which consists of several spatially discontinuous pharma-motifs. A pharma-interface can be described as follows: (1) A pharma-interface is a conserved binding interface of multiple proteins (called polypharmacological targets) which share similar interfaces and are often inhibited by the same compound; (2) A pharma-interface consists of a set of pharma-motifs; (3) A pharma-motif is a short conserved peptide forming a specific sub-interface with interacting residues and specific physico-chemical properties. Figure 1 shows the details of our method to identify a pharma-interface and pharma-motifs from a given complex by the following steps.

#### Step 1: Search candidates of polypharmacological targets

To find the candidates of the polypharmacological targets of a query protein, we firstly transformed the 3D protein structure of a query complex (i.e. protein and its binding ligand) into a 1D sequence with 23 states of a structural alphabet by using in-house tool 3D-BLAST (Figure 1A) [13,14]. We then identified the binding segments in the interface of the query complex. For each segment, its corresponding 1D structural alphabet sequence was used for rapidly searching structural alphabet sequence database (SADB) by using 3D-BLAST and a list of similar protein structures of the query binding segment was returned within several seconds. For the tolerance of the flexible binding environment, the proteins matching more than 75% query segments are considered as the candidates.

Here, we identified a binding segment (≥ 15 amino acids) of a given complex by using a contact residue as a central point to extend seven amino acids forward and backward. An amino acid is considered as a contact residue if the distance between any atom of this residue and the atoms of the binding ligand is less than 4Å. Any of two binding segments with at least one overlapped amino acid are merged into a single one. As a result, the binding environment of a given complex is characterized by spatially discontinuous binding segments.

Next, SRPmotif utilizes 3D-BLAST, a fast protein structure database search, to detect the candidate proteins (i.e. polypharmacological targets) with similar binding segments. 3D-BLAST encodes the 3D structures into a serials of structural alphabets using κ and α angles defined in the DSSP program [17]. Based on the (κ, α) plot of 674 structural pairs and a nearest neighbour clustering, a 23-state structural alphabet represents the profiles of most 3D fragments [14]. A BLOSUM-like substitution matrix, called structural alphabet substitution matrix (SASM) was developed by using a method similar to that used to construct BLOSUM62 [18] based on 674 structural protein pairs. 3D-BLAST searches the SADB using this SASM to quickly discover homologous structures of a query protein.

#### Step 2: Establishment of the pharma-interface

In this step, the polypharmacological targets were determined and used to construct pharma-interface. To determine the polypharmacological targets through the candidate proteins identified in the step 1, a structural alignment tool (DALI [16]) was utilized to precisely locate the possible binding environment in these candidate protein structures (Figure 1B). The DALI program is a structural alignment tool based on contact similarity patterns. Discontinuous binding segments are aligned to a candidate protein and the value of root mean square deviation (RMSD) between all binding segments and a candidate protein is calculated. To estimate opened gaps in a structural alignment, the value of aligned ratio of a binding segment is measured. The aligned ratio is defined below:

(1) $$aligned\;ratio=\frac{number\;of\;aligned\;residues\;of\;candidates}{number\;of\;residues\;in\;queried\;binding\;segment}$$

The range of aligned ratio is from 0 to 1. The RMSD value and the average aligned ratio of discontinuous binding segments are used to evaluate the structure similarity between a query and the candidate proteins. Here, a candidate protein is considered as a polypharmacological target when the value of RMSD is less than 2.0Å and the aligned ratio is great than 0.85. Consequently, a pharma-interface is constructed based on the conserved binding interfaces of polypharmacological targets.

#### Step 3: Characterization of pharma-motifs

The pharma-interface is composed of a set of pharma-motifs, short conserved peptides with interacting residues, to form the binding environment. According to the structure-based multiple sequence alignment (MSA) of the pharma-interfaces of a query, pharma-motifs could be characterized as 1D sequence patterns with the pattern syntax of PROSITE database [1] (Figure 1C).

In order to encode 1D sequence patterns of pharma-motifs, structure-based MSAs are used to measure the sequence conservation. For the position *i *in a MSA, we used Rate4Site algorithm [19,20] to evaluate a conserved score (*S_pi_*) by computing the relative evolutionary rate. The range of *S_pi _*is from 1 to 9 and a position with score of 9 is the most conserved.

In PROSITE syntax, a position labeled as alphabet 'x' means that any kinds of amino acids can occur in this position. Based on 1293 motifs of PROSITE database (release 20.67 of 02-Nov-2010), the average *S_pi _*of encode 'x' is 3.67. Here, a position of pharma-motif is encoded as alphabet 'x' when *S_pi _*is less than 4. Finally, 1D sequence patterns of pharma-motifs are characterized.

### Non-redundant protein-ligand complex dataset

To verify the pharma-interfaces involving key interacting residues and functional sites, we collected a protein-ligand complex dataset which contains 282 FDA approved drugs recorded in DrugPort of PDBsum [21]. We eliminated some protein-ligand complexes based on below criteria: (1) the protein structure is an incomplete structure or a theoretical model; (2) the co-crystal ligand is metal; (3) the number of heavy atoms of ligand is less than six heavy atoms; (4) the co-crystal ligand interacts with multiple protein chains; (5) proteins have greater than 25% sequence identity with others or redundant co-crystal ligands. Final, we selected 89 non-redundant protein-ligand complexes (called FDA89) with structure-based classifications (i.e. SCOP [22] and CATH [23]) and all of the proteins are recorded in the UniProt database [24] (Table S1 in additional file 1).

### Evaluation measures

For evaluating the performance of our method for predicting polypharmacological targets, the recall and precision were calculated. Here, we used biological process (BP) and molecular function (MF) of the Gene Ontology (GO) [25] to annotate polypharmacological targets. In addition, the structural classifications, SCOP (version 1.75) and CATH (version 3.4), were also used to annotate the polypharmacological targets. The recall and precision are defined in the following:

(2) $$recall=\frac{true\;positives}{true\;positives+false\;negatives}$$

(3) $$precision=\frac{true\;positives}{true\;positives+false\;positives}$$

where true positives are predicted as the polypharmacological targets of a query protein and have the same annotation with the query protein; false negatives are predicted as not polypharmacological targets of a query protein and have the same annotation with the query protein; false positives are predicted as the polypharmacological targets of a query protein, but the annotations are different from the query protein.

## Results and discussion

### Evaluations of SRPmotif

**Figure 2:**
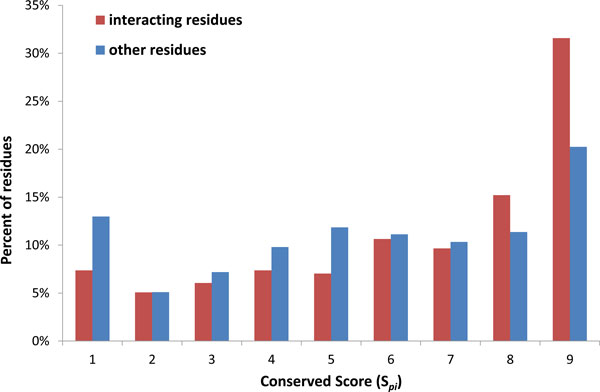
**Conserved score (*S_pi_*) of the residues in the pharma-motifs**. The conserved scores (*S*_*pi*_) of 56.5% interacting residues (red) and 41.9% other residues (blue) in the pharma-motifs are more than 7.0.

In the FDA89 set, 356 pharma-motifs could be identified and the average number of pharma-motifs for each protein-ligand complex is four. To examine whether the pharma-motifs are similar with the PROSITE motifs, a pharma-motif is considered consistent with a PROSITE motif when the intersections of two patterns (i.e. overlapped ratio) are greater than the cut-off (a range of values between 10% and 100%) of the pharma-motif or the PROSITE motif (Figure S1 in additional file 1). The overlapped ratio is defined as the maximum value between the length of the aligned pattern divided by the length of the pharma-motif and that of the PROSITE motif. The result shows that the percentage of overlapped motifs decreases significantly if the overlapped ratio ≥ 50%. Here, the cut-off is set to 50%. Among 66 PROSITE motifs in the FDA89 set, our derived pharma-motifs are consistent with 65% (43/66) PROSITE motifs. Moreover, we compared the evolutionary conservation of the interacting residues with other residues. Figure 2 shows the distributions of the conservation score (S*_pi_*) between the interacting residues and other residues in 356 pharma-motifs. For highly conservation (*S_pi _*≥ 7), the *S_pi _*values of the interacting residues (56.5%) were significantly higher than those of other residues (41.9%). In addition, 31.6% interacting residues were conserved with the highest *S_pi _*values. These results show that the interacting residues involved in pharma-motifs are highly conserved and encoded as conserved pattern.

Precision and recall rates were utilized to assess the similarity of biological functions (i.e. BP and MF) and structural classifications (i.e. SCOP and CATH) between polypharmacological targets and their query proteins of protein-ligand complexes. Based on BP and MF annotations, the precision rates are 81.1% and 92.7%, respectively (Table 1). These experimental results show that polypharmacological targets not only are involved in the similar cellular process but also perform similar biological functions. Moreover, the precision rates are 55.2% and 79.7% for SCOP and CATH, respectively (Table 1). In the above results, the polypharmacological targets without annotations are considered as negatives. The precision rates are more than 90% when the polypharmacological targets without any annotations are removed. The high precision rates show that polypharmacological targets of each protein-ligand complex are usually recorded in the same structure family.

**Table 1:** Precision and recall of polypharmacological targets using gene ontology (GO) annotations or structure classifications

GO annotations or structure classification	Precision (%)	Recall (%)	Number of true positives	Number of polypharmacological targets	Number of positives in database
Biological process	81.1	31.5	9702	11956	30810
Molecular function	92.7	19.9	11089	11956	55662
SCOP	55.2	29.7	6596	11956	22213
CATH	79.7	12.8	9533	11956	74294

However, the results with low recall rates may imply that proteins with the same annotation (i.e. biological function or the structure family) sometimes have the key difference in protein-ligand binding environments. For example, viral neuraminidase (NA) of influenza virus is a drug target for prevention of influenza infection and has several homologous proteins (e.g. Sialidase 2 (NEU2)) in the human genome. Both of NA and NEU2 are a type of glycoside hydrolase enzymes (Enzyme Commission number (EC) 3.2.1.18). NA (PDB code: 1NNC[26], chain A) and NEU2 (PDB code: 2F0Z, chain A) have crystal structures with the same drug, Zanamivir (ZMR, listing name Relenza), and are classified as identical structure family in SCOP (b.68.1.1) and CATH (2.120.10.10).

The NEU2 or NA is not the polypharmacological target of NA-ZMR complex or NEU2-ZMR complex, respectively. After observing the binding environments of NA-ZMR and NEU2-ZMR complexes, the interacting residues and identified binding segments of two complexes are different (RMSD of binding segments is 3.8). Previous experiment results showed that NA is inhibited by ZMR in nM level (H1N1: 1.56 nM, H3N2: 2.66 nM, H5N3: 3.97 nM) while NEU2 is inhibited by ZMR in μM level (16.4 μM) [27]. The proteins in the same structure family (i.e. SCOP and CATH) might be subdivided to many sub-families by SRPmotif due to different binding environments. Therefore, the SRPmotif method is sensitive and precise to identify the pharma-motifs for describing the binding environment between protein and ligand.

### Example analysis: Isoleucyl-tRNA synthetase and Mupirocin

**Figure 3:**
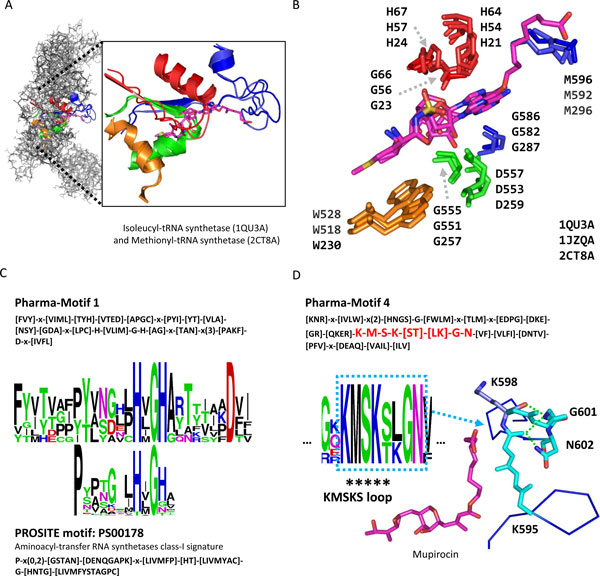
**The pharma-interface and pharma-motifs of Isoleucyl-tRNA synthetase-Mupirocin complex**. (A) The pharma-motif superimposition between Isoleucyl-tRNA synthetase and its polypharmacological target, Methionyl-tRNA synthetase, is well aligned. These two proteins are belonging to the different structure families and the pharma-motifs are the color segments. (B) Interacting residues are highly conserved in these pharma-motifs. The ligands are colored by magenta. (C) Pharma-motif 1 and the corresponding PROSITE motif, PS00178. (D) Pharma-motif 4 is the ATP binding motif and involves KMSKS loop with two conserved residues (G601 and N602). These two residues stabilize the KMSKS loop by using hydrogen bonds (green line) with residue K598.

Figure 3 shows that the pharma-interface and pharma-motifs by using the Isoleucyl-tRNA synthetase (ileS) of *Staphylococcus aureus *and Mupirocin complex (PDB code: 1QU3[28], chain A) as the query template. Bacterial ileS is involved in the incorporation of isoleucine into bacterial proteins. Mupirocin is an antibiotic originally isolated from *Pseudomonas fluorescens *NCIMB 10586. It is also selective binding to bacterial ileS to achieve bacteriostasis at low concentrations and bactericidal effect at high concentrations against Gram-positive bacteria. For this protein-ligand complex, SRPmotif yielded 20 polypharmacological targets from the PDB database (Table 2). These polypharmacological targets have similar functions but belong to different ligases forming aminoacyl-tRNA (EC 6.1.1.*). SRPmotif also detected a protein (i.e. Methionyl-tRNA synthetase (metG)) recorded in different structure family with ileS. Figure 3A shows that the superimposition of binding environments between proteins ileS and metG (PDB code: 2CT8[29], chain A). In the identified pharma-motifs, the interacting residues of query complex are highly conserved and spatially closed (Figure 3B). For example, the residues H64, G66, and H67 of Pharma-Motif 1 in ileS (PDB code: 1QU3) are encoded as conserved positions. Previous study indicated that H-x-G-H (HxGH loop) involved in Pharma-Motif 1 was a signature sequence in class-I aminoacyl-transfer RNA synthetases [12]. Additionally, one PROSITE motif, aminoacyl-transfer RNA synthetases class-I signature (PS00178), is covered by Pharma-Motif 1. The pattern encodings are consistent with the PS00178, especially in interacting residues, such as residues H64 and G66 of HxGH loop (Figure 3C).

**Table 2:** Polypharmacological targets identified by using Isoleucyl-tRNA synthetase-Mupirocin complex as the query

PDB Code	UniProt AC	EC number	Ligand	SCOP	CATH	Species	Gene Name	Description	RMSD	AR	SI
1qu3A	P41972	6.1.1.5	MRC, ZN	a.27.1.1, b.51.1.1, c.26.1.1	1.10.730.10, 3.90.740.10	Staphylococcus aureus	ileS	Isoleucyl-tRNA synthetase	0	1	100
1ffyA	P41972	6.1.1.5	MRC, ZN	a.27.1.1, b.51.1.1, c.26.1.1	-	Staphylococcus aureus	ileS	Isoleucyl-tRNA synthetase	0.4	1	100
1qu2A	P41972	6.1.1.5	MRC, ZN	a.27.1.1, b.51.1.1, c.26.1.1	-	Staphylococcus aureus	ileS	Isoleucyl-tRNA synthetase	0.4	1	100
1ileA	P56690	6.1.1.5	ZN	a.27.1.1, b.51.1.1, c.26.1.1	1.10.730.10, 3.40.50.620, 3.90.740.10	Thermus thermophilus	ileS	Isoleucyl-tRNA synthetase	1.4	1	31.8
1jzqA	P56690	6.1.1.5	ILA, ZN	a.27.1.1, b.51.1.1, c.26.1.1	1.10.730.10, 3.40.50.620, 3.90.740.10	Thermus thermophilus	ileS	Isoleucyl-tRNA synthetase	1.4	1	31.8
1jzsA	P56690	6.1.1.5	MRC, ZN	a.27.1.1, b.51.1.1, c.26.1.1	1.10.730.10, 3.40.50.620, 3.90.740.10	Thermus thermophilus	ileS	Isoleucyl-tRNA synthetase	1.4	1	31.8
2d5bA	P23395	6.1.1.10	ZN	a.27.1.1, c.26.1.1	1.10.730.10, 3.40.50.620	Thermus thermophilus	metG	Methionyl-tRNA synthetase	1.7	1	26.8
2d54A	P23395	6.1.1.10	ZN	a.27.1.1, c.26.1.1	1.10.730.10, 3.40.50.620	Thermus thermophilus	metG	Methionyl-tRNA synthetase	1.7	1	26.8
1a8hA	P23395	6.1.1.10	ZN	a.27.1.1, c.26.1.1	1.10.730.10, 2.170.220.10, 3.40.50.620	Thermus thermophilus	metG	Methionyl-tRNA synthetase	1.7	1	26.8
1woyA	P23395	6.1.1.10	ZN	a.27.1.1, c.26.1.1	1.10.730.10, 2.170.220.10, 3.40.50.620	Thermus thermophilus	metG	Methionyl-tRNA synthetase	1.8	1	26.8
1li5A	P21888	6.1.1.16	ZN	a.27.1.1, c.26.1.1	3.40.50.620	Escherichia coli	cysS	Cysteinyl-tRNA synthetase	1.9	0.99	30.5
1li7A	P21888	6.1.1.16	CYS, ZN	a.27.1.1, c.26.1.1	3.40.50.620	Escherichia coli	cysS	Cysteinyl-tRNA synthetase	1.9	0.99	30.5
1li7B	P21888	6.1.1.16	CYS, ZN	a.27.1.1, c.26.1.1	3.40.50.620	Escherichia coli	cysS	Cysteinyl-tRNA synthetase	1.9	0.95	30.5
1li5B	P21888	6.1.1.16	ZN	a.27.1.1, c.26.1.1	3.40.50.620	Escherichia coli	cysS	Cysteinyl-tRNA synthetase	2	0.95	30.5
1wkbA	O58698	6.1.1.4	SO4	-	1.10.730.10, 3.40.50.620, 3.90.740.10	Pyrococcus horikoshii	leuS	Leucyl-tRNA synthetase	2	0.99	22.3
1wz2A	O58698	6.1.1.4	-	-	1.10.730.10, 3.40.50.620, 3.90.740.10	Pyrococcus horikoshii	leuS	Leucyl-tRNA synthetase	2	0.99	21.7
1wz2B	O58698	6.1.1.4	-	-	1.10.730.10, 3.40.50.620, 3.90.740.10	Pyrococcus horikoshii	leuS	Leucyl-tRNA synthetase	2	0.99	21.7
2ct8A	O67298	6.1.1.10	MSP	-	3.40.50.620	Aquifex aeolicus	metG	Methionyl-tRNA synthetase	2	0.99	25.2
2ct8B	O67298	6.1.1.10	MSP	-	3.40.50.620	Aquifex aeolicus	metG	Methionyl-tRNA synthetase	2	0.99	25.2
2csxA	O67298	6.1.1.10	-	-	3.40.50.620	Aquifex aeolicus	metG	Methionyl-tRNA synthetase	2	0.99	25.2

Pharma-Motif 4 contains a highly conserved region (i.e. K-M-S-K-[ST]-[LK]-G-N) which is not recoded in PROSITE database. This region includes the KMSKS loop (K-M-S-K-[ST]) and one of residues (i.e. K598) is important to ATP binding [12,30] (Figure 3B). The conserved residues, G601 and N602, interact to KMSKS loop by hydrogen bonds (Figure 3D). It suggests that two conserved residues could stabilize the structure of KMSKS loop and enable K598 to bind ATP. In addition, several residues of derived pharma-motifs are highly conserved to stabilize the structure or protein-ligand interaction, such as D75 in Pharma-Motif 1 (Figure 3C) and G586 in Pharma-Motif 4 (Figure 3B). These results show that SRPmotif could recognize the proteins with similar functions and binding motifs, even are recoded in the different structure families. Furthermore, the pharma-motifs include the conserved interacting residues on functional sites for studying protein functions and binding mechanisms.

### Example analysis: tyrosine-protein kinase KIT and Imatinib

Protein kinases play important roles in cell growth and signal transduction. Protein KIT is one of tyrosine-protein kinases and a kind of receptor of stem cell factors. Imatinib (list name: Gleevec or Glivec) is an inhibitor for tyrosine-protein kinases, including KIT, ABL1, ABL2, and PDGFR. By blocking ATP binding site, Imatinib inhibits tyrosine-protein kinases and is approved to treat chronic myelogenous leukemia and gastrointestinal stromal tumor. Here, we used tyrosine-protein kinase KIT (PDB code: 1T46[31], chain A) and Imatinib complex as query to search against the PDB database and construct the pharma-interface and pharma-motifs.

**Figure 4:**
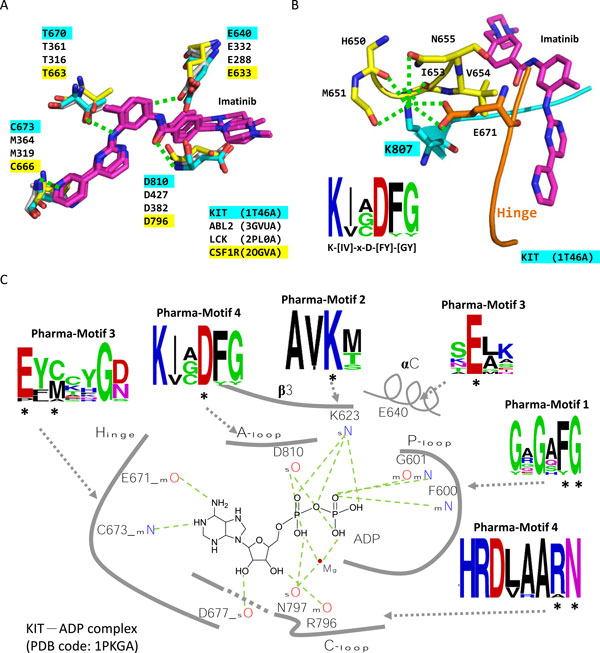
**The pharma-interface and pharma-motifs of the tyrosine-protein kinase KIT-Imatinib complex**. (A) The four residues (E640, T670, C673 and D810) of KIT protein (cyan) form hydrogen bonds (green dashed lines) with Imatinib (magenta) and are conserved in this family. (B) The conserved residue K807 plays an important role to stabilize the kinase structures. (C) The conserved residues in these pharma-motifs are related to phosphorylation of KIT. Asterisks mark the residues interacting with the ATP with hydrogen bonds.

The polypharmacological targets of KIT-Imatinib complex consists of 25 tyrosine-protein kinases. SRPmotif recognizes the protein CSF1R, which has no crystal structures of Imatinib binding. Figure 4A presents the residues interacted with Imatinib by hydrogen bonds in three structures having co-crystal Imatinib (PDB code: 1T46, chain A, PDB code: 3GVU, chain A, and PDB code: 2PL0[32], chain A). These residues (e.g. E640, T670, C673 and D810) are conserved in sequence and structure of protein CSF1R (PDB code: 2OGV[33], chain A). Previous experiment has also showed that CSF1R is inhibited by Imatinib in 19nM [34]. It suggests that Imatinib could be possible to treat acute myeloid leukemia through inhibiting CSF1R.

The pharma-motifs, Pharma-Motif 1 and 2, have overlapped with the identical PROSITE motifs (Protein kinases ATP-binding region signature, PS00107). The highly conserved regions, [LIQ]-[GY]-x-G-[AQSH]-[FY]-G-x-V and [VC]-A-[VI]-K, are similar to PS00107 (Figure S2 in additional file 1, upper side). It also involves to the GxGxFG motif in P-loop of a kinase [35], which is related to interact with phosphate of ATP. The Pharma-Motif 4 contains two highly conserved regions, [IV]-H-R-D-[LV]-[AR]-A-[RA]-N and K-[IV]-x-D-[FY]-[GY]. The former is alike to the known motif (Tyrosine protein kinases specific active-site signature, PS00109) and is related to kinase catalysis, named the catalytic loop in a kinase (Figure S2 in additional file 1, bottom side). Previous study has showed that the conserved aspartic acid in the catalytic loop may be important in positioning the substrate hydroxyl for in-line nucleophilic attack [36] (Figure 4C). The latter has been reported as conserved peptide, DFG motif [35], which is the beginning of activation loop of a kinase. The conformation of DFG motif is relevant to kinase activation and the conserved aspartic acid binds directly to the magnesium ion cofactor orienting the γ-phosphate group of ATP for transfer (Figure 4C).

We also observed the conserved lysine in the front of DFG motif in Pharma-Motif 4. Figure 4B shows that the residue K807 (sequence number in protein 1T46) interacts many neighbouring residues in space by hydrogen bonds, such as E671 in Hinge region, M651, I653, and N655. The residue V654 close to K807 usually interacts with inhibitors. It suggests that conserved K807 could compact the kinases structure to keep the interaction of V654 and inhibitors. Figure 4C shows that these conserved residues of the identified pharma-motifs are related to phosphorylation. For example, the conserved lysine in Pharma-Motif 2 is helpful for stabilizing γ- and α-phosphate groups of ATP. The conserved glutamic acid in Pharma-Motif 3 is helpful for stabilizing γ-phosphate group of ATP. These results show that pharma-motifs involved important interacting residues and conserved positions of the pharma-motifs could play important roles in protein function and binding mechanisms.

### Computational time

SRPmotif utilizes structural alphabets and 3D-BLAST for searching similar structure segments within 1 minute on average. During the construction of a pharma-interface and pharma-motifs, the computational time for a query complex depends on the number of polypharmacological candidates and hit targets. On average, for a query complex, such as Isoleucyl-tRNA synthetase-Mupirocin complex, SRPmotif searches the structure database and characterizes the pharma-interface and pharma-motifs within 5 minutes. In some cases, SRPmotif identified pharma-motifs from over hundreds of target proteins within 10 minutes. These tests were conducted on a Linux platform with Intel^® ^Quad-core processors and 8G RAM.

## Conclusions

SRPmotif is able to identify similar pharma-interfaces and pharma-motifs sharing similar binding environments for polypharmacological targets by rapidly searching against the protein structure database. The experimental results demonstrate that the identified pharma-motifs would comprise key residues and important functional sites. Pharma-motifs describe the conservations of binding environments for polypharmacological targets and provide clues for discovering new sequence-based motifs for predicting protein functions from protein sequence database. We believe that SRPmotif is useful for elucidating protein functions, protein-ligand interactions, and drug discovery.

## Competing interests

The authors declare that they have no competing interests.

## Authors' contributions

YYC, LZC, and JMY conceived and designed the experiments. YYC, LZC, and JMY implemented the materials/analysis programs. YYC, CTL, LZC, and JMY performed the experiments and analyzed the data. YYC, CYL, CTL, KCH, and JMY wrote the paper.

## Supplementary Material

Additional file 1 **Supplementary figures and table**. Click here for file
