# Supplementary material for: Space-related pharma-motifs for fast search of protein binding motifs and polypharmacological targets
Source: BMC Genomics. 2012 Dec 7;13(Suppl 7):S21. doi: 10.1186/1471-2164-13-S7-S21 (PMC3521469; doi:10.1186/1471-2164-13-S7-S21)
Supplement: Additional file 1 — Supplementary figures and table. [file 1471-2164-13-S7-S21-S1.pdf]

Additional files

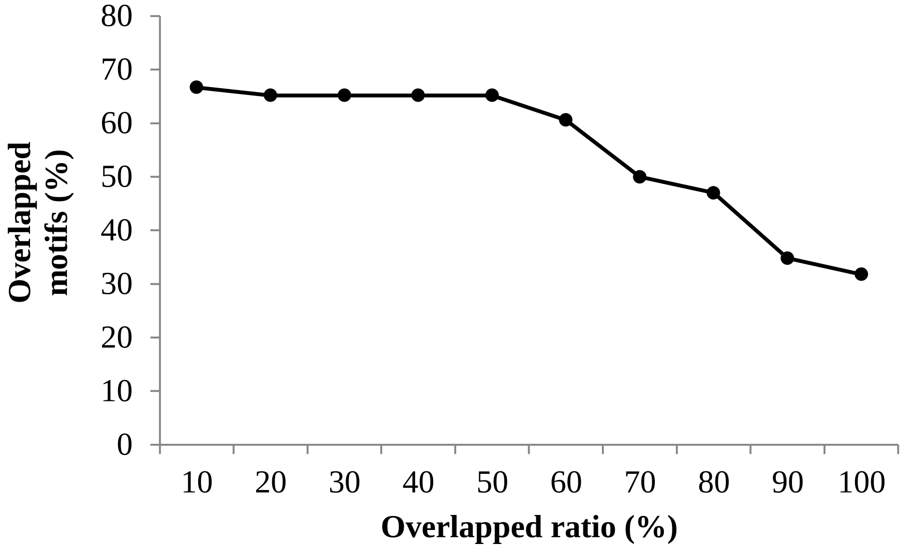

Supplementary Figure S1 – The relationship between the percentage of overlapped motifs and the cut-off of overlapped ratio.

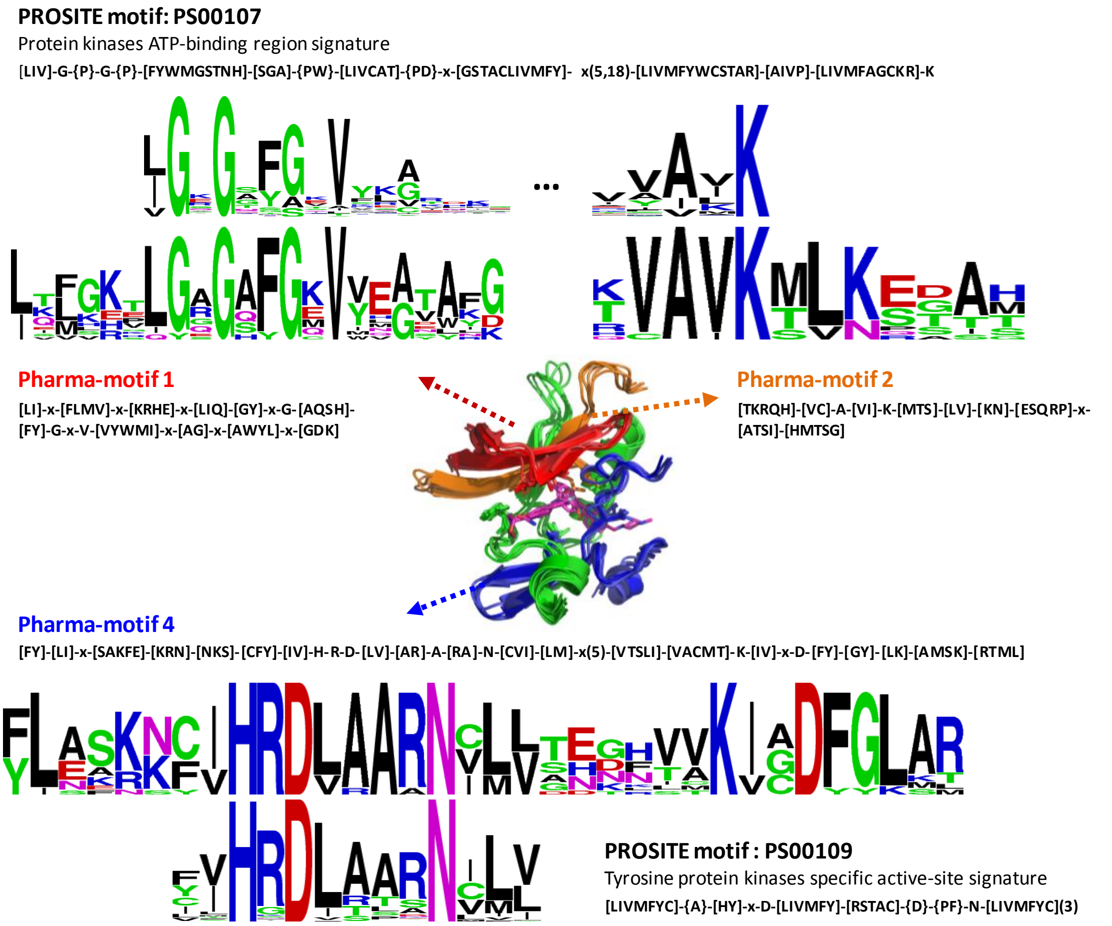

Supplementary Figure S2 - The pharma-motifs of tyrosine-protein kinase KIT －Imatinib complex and the corresponding PROSITE motifs.
Four pharma-motifs are identified using tyrosine-protein kinase KIT and Imatinib complex as the query. Among these four pharma-motifs, two motifs are recorded in PROSITE, PS00107 and PS00109. Pharma-motifs 1 and 2 (upper) correspond to PROSITE motif (PS00107) and Pharma-motif 4 (bottom) forms a highly conserved region (called DFG motif) corresponds to PROSITE motif (PS00109).

Supplementary Table S1 - List of 89 non-redundant protein-ligand complexes

PDB Code	Ligand	Uniprot AC	EC number	SCOP	CATH	Gene Name	Description
1a4gA	ZMR	P27907	3.2.1.18	b.68.1.1	2.120.10.10	NA	Neuraminidase
1a4lC	DCF	P03958	3.5.4.4	c.1.9.1	3.20.20.140	Ada	Adenosine deaminase
1ax9A	EDR	P04058	3.1.1.7	c.69.1.1	3.40.50.1820	ache	Acetylcholinesterase
1azmA	AZM	P00915	4.2.1.1	b.74.1.1	3.10.200.10	CA1	Carbonic anhydrase 1
1b3nA	CER	P0AAI5	2.3.1.179	c.95.1.1	3.40.47.10	fabF	3-oxoacyl-[acyl-carrier-protein] synthase 2
1bkfA	FK5	P62942	5.2.1.8	d.26.1.1	3.10.50.40	FKBP1A	Peptidyl-prolyl cis-trans isomerase FKBP1A
1bsxA	T3	P10828	-	a.123.1.1	1.10.565.10	THRB	Thyroid hormone receptor beta
1bt5A	IM2	P62593	3.5.2.6	e.3.1.1	3.40.710.10	bla	Beta-lactamase TEM
1bzfA	TMQ	P00381	1.5.1.3	c.71.1.1	3.40.430.10	folA	Dihydrofolate reductase
1cetA	CLQ	Q27743	1.1.1.27	c.2.1.5, d.162.1.1	3.40.50.720, 3.90.110.10	-	L-lactate dehydrogenase
1cqeA	FLP	P05979	1.14.99.1	a.93.1.2, g.3.11.1	1.10.640.10, 2.10.25.10	PTGS1	Prostaglandin G/H synthase 1
1ctrA	TFP	P62158	-	a.39.1.5	1.10.238.10	CALM1	Calmodulin
1dyrA	TOP	P16184	1.5.1.3	c.71.1.1	3.40.430.10	-	Dihydrofolate reductase
1dzmB	BZM	P81245	-	b.60.1.1	2.40.128.20	-	Odorant-binding protein
1ei6C	PPF	Q51782	3.11.1.2	c.76.1.4	3.30.1360.110, 3.40.720.10	phnA	Phosphonoacetate hydrolase
1f5lA	AMR	P00749	3.4.21.73	b.47.1.2	2.40.10.10	PLAU	Urokinase-type plasminogen activator
1fb7A	ROC	P04585	2.7.7.49, 3.1.26.13, 3.4.23.16, 2.7.7.7, 3.1.13.2	b.50.1.1	2.40.70.10	gag-pol	Gag-Pol polyprotein
1fcnB	LOR	P00811	3.5.2.6	e.3.1.1	3.40.710.10	ampC	Beta-lactamase
1gsfC	EAA	P08263	2.5.1.18	a.45.1.1, c.47.1.5	1.20.1050.10, 3.40.30.10	GSTA1	Glutathione S-transferase A1
1h61A	PDN	P71278	-	c.1.4.1	3.20.20.70	-	-
1h8yA	MER	Q51400	-	e.3.1.1	3.40.710.10	-	-
1i1eA	DM2	P10844	3.4.24.69	b.29.1.6, b.42.4.2, d.92.1.7, h.4.2.1	1.20.1120.10, 2.60.120.200, 2.80.10.50, 3.90.1240.10	botB	Botulinum neurotoxin type B
1i2wA	CFX	P00808	3.5.2.6	e.3.1.1	3.40.710.10	penP	Beta-lactamase
1ihiA	IU5	P52895	1.1.1.213, 1.3.1.20	c.1.7.1	3.20.20.100	AKR1C2	Aldo-keto reductase family 1 member C2
1ituA	CIL	P16444	3.4.13.19	c.1.9.7	3.20.20.140	DPEP1	Dipeptidase 1
1j3jA	CP6	P13922	1.5.1.3, 2.1.1.45	c.71.1.1	3.40.430.10	-	Bifunctional dihydrofolate reductase-thymidylate synthase
1jb0B	PQN	P0A407	-	f.29.1.1	1.20.1130.10	psaB	Photosystem I P700 chlorophyll a apoprotein A2
1jqeA	QUN	P50135	2.1.1.8	c.66.1.19	3.40.50.150	HNMT	Histamine N-methyltransferase
1jr1A	MOA	P12269	1.1.1.205	c.1.5.1, d.37.1.1	3.20.20.70	IMPDH2	Inosine-5'-monophosphate dehydrogenase 2
1ki3A	PE2	P03176	2.7.1.21	c.37.1.1	3.40.50.300	TK	Thymidine kinase

Supplementary Table S1 - 89 non-redundant protein-ligand complexes (Cont'd)

PDB Code	Ligand	Uniprot AC	EC number	SCOP	CATH	Gene Name	Description
1klmA	SPP	P04585	3.4.23.16, 3.1.26.13, 3.1.13.2, 2.7.7.7, 2.7.7.49	c.55.3.1, e.8.1.2	3.10.10.10, 3.30.70.270, 3.30.420.10	gag-pol	Gag-Pol polyprotein
1lhvA	NOG	P04278	-	b.29.1.4	2.60.120.200	SHBG	Sex hormone-binding globulin
1m17A	AQ4	P00533	2.7.10.1	d.144.1.7	1.10.510.10, 3.30.200.20	EGFR	Epidermal growth factor receptor
1m2xC	MCO	O08498	3.5.2.6	d.157.1.1	3.60.15.10	blaB1	Carbapenem-hydrolyzing beta-lactamase BlaB-1
1m2zA	DEX	P04150	-	a.123.1.1	1.10.565.10	NR3C1	Glucocorticoid receptor
1m4dA	TOY	P0A5N0	-	d.108.1.1	3.40.630.30	aac	Aminoglycoside 2'-N-acetyltransferase
1m9jA	CLW	P29474	1.14.13.39	d.174.1.1	3.90.440.10, 3.90.1230.10	NOS3	Nitric oxide synthase, endothelial
1maaA	DME	P21836	3.1.1.7	c.69.1.1	3.40.50.1820	Ache	Acetylcholinesterase
1n0sA	FLU	P09464	-	b.60.1.1	2.40.128.20	-	Bilin-binding protein
1nd4A	KAN	P00552	2.7.1.95	d.144.1.6	3.30.200.20, 3.90.1200.10	neo	Aminoglycoside 3'-phosphotransferase
1nnfA	EDT	P35755	-	c.94.1.1	3.40.190.10	fbpA	Iron-utilization periplasmic protein
1nx9C	AIC	Q8VRK8	3.1.1.43	b.18.1.13, c.69.1.21	1.10.3020.10, 2.60.120.260, 3.40.50.1820	-	-
1ohpC	ESR	P00947	5.3.3.1	d.17.4.3	3.10.450.50	ksi	Steroid Delta-isomerase
1p5zB	AR3	P27707	2.7.1.74	c.37.1.1	3.40.50.300	DCK	Deoxycytidine kinase
1p7rA	NCT	P00183	1.14.15.1	a.104.1.1	1.10.630.10	camC	Camphor 5-monooxygenase
1pbcA	BHA	P00438	1.14.13.2	c.3.1.2, d.16.1.2	3.30.9.10, 3.50.50.60	pobA	P-hydroxybenzoate hydroxylase
1qcaA	FUA	P00484	2.3.1.28	c.43.1.1	3.30.559.10	cat3	Chloramphenicol acetyltransferase 3
1qknA	RAL	Q62986	-	a.123.1.1	1.10.565.10	Esr2	Estrogen receptor beta
1qu3A	MRC	P41972	6.1.1.5	a.27.1.1, b.51.1.1, c.26.1.1	1.10.730.10, 3.90.740.10	ileS	Isoleucyl-tRNA synthetase
1rkwA	PNT	P0A0N4	-	a.4.1.9, a.121.1.1	1.10.10.60, 1.10.357.10	qacR	HTH-type transcriptional regulator qacR
1rtsA	D16	P45352	2.1.1.45	d.117.1.1	3.30.572.10	Tyms	Thymidylate synthase
1rxcK	URF	P12758	2.4.2.3	c.56.2.1	3.40.50.1580	udp	Uridine phosphorylase
1s14A	NOV	P20083	-	d.122.1.2	3.30.565.10	parE	DNA topoisomerase 4 subunit B
1s9pA	DES	P62508	-	a.123.1.1	1.10.565.10	ESRRG	Estrogen-related receptor gamma
1sqnA	NDR	P06401	-	a.123.1.1	1.10.565.10	PGR	Progesterone receptor
1t46A	STI	P10721	2.7.10.1	d.144.1.7	1.10.510.10, 3.30.200.20	KIT	Mast/stem cell growth factor receptor
1tbfA	VIA	O76074	3.1.4.35	a.211.1.2	1.10.1300.10	PDE5A	cGMP-specific 3',5'-cyclic phosphodiesterase
1td7A	NFL	P60045	3.1.1.4	a.133.1.2	1.20.90.10	-	Phospholipase A2 isoform 3 (Fragment)
1tlmA	MIL	P02766	-	b.3.4.1	2.60.40.180	TTR	Transthyretin
1tubB	TXL	P02554	-	c.32.1.1, d.79.2.1	1.10.287.600, 3.30.1330.20, 3.40.50.1440	-	Tubulin beta chain

Supplementary Table S1 - 89 non-redundant protein-ligand complexes (Cont'd)

PDB Code	Ligand	Uniprot AC	EC number	SCOP	CATH	Gene Name	Description
1uwhA	BAX	P15056	2.7.11.1	d.144.1.7	1.10.510.10, 3.30.200.20	BRAF	Serine/threonine-protein kinase B-raf
1v3qE	2DI	P00491	2.4.2.1	c.56.2.1	3.40.50.1580	PNP	Purine nucleoside phosphorylase
1v8bC	ADN	P50250	3.3.1.1	c.2.1.4, c.23.12.3	3.40.50.720, 3.40.50.1480	PFE1050w	Adenosylhomocysteinase
1x70A	715	P27487	3.4.14.5	b.70.3.1, c.69.1.24	2.140.10.30, 3.40.50.1820	DPP4	Dipeptidyl peptidase 4
1xotB	VDN	Q07343	3.1.4.17	a.211.1.2	1.10.1300.10	PDE4B	cAMP-specific 3',5'-cyclic phosphodiesterase 4B
1xr3B	ISZ	P16544	-	c.2.1.2	3.40.50.720	actIII	Putative ketoacyl reductase
1y7iB	SAL	Q6RYA0	-	c.69.1.20	3.40.50.1820	-	-
1y8eA	SVR	P68638	-	g.18.1.1	2.10.70.10	C3L	Complement control protein
1zzqA	MTL	P29476	1.14.13.39	d.174.1.1	3.90.440.10, 3.90.1230.10	Nos1	Nitric oxide synthase, brain
2a3rA	LDP	P50224	2.8.2.1	c.37.1.5	3.40.50.300	SULT1A3	Sulfotransferase 1A3/1A4
2a69M	RPT	Q8RQE9	2.7.7.6	e.29.1.1	2.30.150.10, 2.40.50.100, 2.40.50.150, 2.40.270.10, 3.90.1100.10, 3.90.1110.10	rpoB	DNA-directed RNA polymerase subunit beta
2b0qA	NMY	P0A3Y5	2.7.1.95	d.144.1.6	3.30.200.20, 3.90.1200.10	aphA	Aminoglycoside 3'-phosphotransferase
2b17A	DIF	P59071	3.1.1.4	a.133.1.2	1.20.90.10	-	Phospholipase A2 VRV-PL-VIIIa
2bxcA	P1Z	P02768	-	a.126.1.1	1.10.246.10	ALB	Serum albumin
2fumC	MIX	P0A5S4	2.7.11.1	d.144.1.7	1.10.510.10, 3.30.200.20	pknB	Serine/threonine-protein kinase pknB
2ij7D	TPF	P0A514	-	a.104.1.1	1.10.630.10	cyp121	Cytochrome P450 121
2inqB	MT1	P0ABQ4	1.5.1.3	c.71.1.1	3.40.430.10	folA	Dihydrofolate reductase
2j0dA	ERY	P08684	1.14.13.32, 1.14.13.67, 1.14.13.97	a.104.1.1	1.10.630.10	CYP3A4	Cytochrome P450 3A4
2jj8D	AZZ	Q9XZT6	2.7.1.145	c.37.1.1	3.40.50.300	dnk	Deoxynucleoside kinase
2nniA	MTK	P10632	1.14.14.1	a.104.1.1	1.10.630.10	CYP2C8	Cytochrome P450 2C8
2o7oA	DXT	P0ACT4	-	a.4.1.9, a.121.1.1	1.10.10.60, 1.10.357.10	tetR	Tetracycline repressor protein class D
2pouA	I7A	P00918	4.2.1.1	b.74.1.1	3.10.200.10	CA2	Carbonic anhydrase 2
2prgA	BRL	P37231	-	a.123.1.1	1.10.565.10	PPARG	Peroxisome proliferator-activated receptor gamma
2qwhA	G39	P03472	3.2.1.18	b.68.1.1	2.120.10.10	NA	Neuraminidase
2v3dA	NBV	P04062	3.2.1.45	b.71.1.2, c.1.8.3	2.60.40.1180, 3.20.20.80	GBA	Glucosylceramidase
3c0zB	SHH	Q8WUI4	3.5.1.98	c.42.1.2	3.40.800.20	HDAC7	Histone deacetylase 7
3cflA	5CH	P24627	-	c.94.1.2	3.40.190.10	LTF	Lactotransferrin
3zncA	BZ1	Q64444	4.2.1.1	b.74.1.1	3.10.200.10	Ca4	Carbonic anhydrase 4
4pahA	LNR	P00439	1.14.16.1	d.178.1.1	1.10.800.10	PAH	Phenylalanine-4-hydroxylase
